# Efforts to implement WHO recommendations on antenatal, intrapartum and postnatal care

**DOI:** 10.2471/BLT.25.294453

**Published:** 2025-09-01

**Authors:** Mercedes Bonet, Maria Barreix, Shuchita Gupta, Tigest Tamrat, Özge Tunçalp, Anayda Portela

**Affiliations:** a UNDP/UNFPA/UNICEF/WHO/World Bank Special Programme of Research, Development and Research Training in Human Reproduction, Department of Sexual and Reproductive Health and Research, World Health Organization, Avenue Appia 20, 1211 Geneva 27, Switzerland.; b Department of Maternal, Newborn, Child and Adolescent Health and Ageing, World Health Organization, Geneva, Switzerland.

Maternal and perinatal morbidity and mortality remain unacceptably high. Many countries will fall short of achieving sustainable development goal targets on maternal and neonatal mortality, resulting in many women and newborns facing life-threatening conditions and sequelae.^1^ In 2024, the World Health Assembly reaffirmed that high-quality antenatal care, intrapartum care and postnatal care are fundamental to the health and well-being of women and their newborns, and critical for reducing preventable maternal, fetal and neonatal deaths and complications.^1^

The World Health Organization (WHO) continuously updates and publishes recommendations for improving maternal and newborn care. These recommendations mainly focus on provision of high-quality care, ensuring a positive experience throughout pregnancy, childbirth and the postnatal period.^2^^–^^4^ They highlight maternal well-being and mental health; prevention and screening strategies; breastfeeding and nurturing care for newborns; better preparation for discharge and transition to self-care and family care in the home after birth; strengthened health systems and workforce; and integrated service delivery. Yet, implementation remains uneven. In many settings, demand for and coverage of high-quality antenatal, intrapartum and postnatal care remains low, and recommended practices are not consistently reflected in local guidelines and clinical practice.^5^ In response to country requests for technical and implementation support, WHO has developed a suite of implementation tools to bridge this gap. These tools support translation of global recommendations into context-appropriate policies, guidelines, programmes and practices. 

Two WHO toolkits aim to facilitate the adoption, adaptation and implementation of routine antenatal, intrapartum and postnatal care recommendations. The *Toolkit for adaptation of the WHO recommendations for a positive pregnancy and postnatal experience*^6^ supports countries in developing or updating national and subnational guidelines, and integrated country-specific intervention packages of antenatal and postnatal care. This toolkit encourages integration of services within existing health systems and alignment with community preferences and legal frameworks, including wider legislation beyond essential services, such as birth registration, parental protections and regulation against the marketing of breastmilk substitutes.^7^ The *Toolkit for implementation of the WHO intrapartum care and immediate postnatal care recommendations in health-care facilities*^8^ offers guidance for facility-level change. This second toolkit draws on implementation and behavioural science to support managers and health workers to identify priority practices and influencing factors, and to apply strategies likely to successfully change behaviours.

Both toolkits were co-developed with stakeholders from policy, programme, clinical and implementation backgrounds at global, regional and country levels. The toolkits orient users through a participatory, stepwise process to identify national or subnational adaptation needs and implementation enablers and barriers, and to select and implement priority actions to improve the demand, uptake and provision of high-quality maternal and newborn health services. Each toolkit includes a user guide with structured steps, and for each step a set of resources to complete baseline assessments and identify obstacles and enablers, summaries of evidence, case examples and recommended indicators aligned with global monitoring frameworks. Although these tools provide specific guidance for essential care during pregnancy, childbirth and the postnatal period, the processes proposed could also be followed when preparing for implementation of recommendations for management of maternal and newborn complications or recommendations related to other health domains.

In addition, digital adaptation kits for antenatal^9^ and postnatal care^10^ are now available to support the integration of WHO’s recommendations into digital systems. These kits enable translation of guidance into electronic platforms, facilitating the implementation of the country-adapted intervention packages. The digital adaptation kits are foundational components in translating WHO recommendations to existing or newly developed digital systems, which in turn support health workers through decision support and longitudinal patient tracking. Digital adaptation kits can also support linkages to other services such as immunization and family planning, as well as birth notifications. A digital adaptation kit for intrapartum care is under development to support quality of care and data flows across the maternal and newborn health continuum.

The way forward depends on international, national and subnational government and stakeholder commitment to improving use and provision of high-quality maternal and newborn health services.^11^ To accelerate progress, countries can adapt and implement WHO’s maternal and newborn care recommendations using these tools. The tools offer a practical path forward, bridging the gap between global guidance, national programmes and local practices, leading to improved health outcomes for women and newborns in the context of universal health coverage and human rights-based approaches. 
